# Strong families: a new family skills training programme for challenged and humanitarian settings: a single-arm intervention tested in Afghanistan

**DOI:** 10.1186/s12889-020-08701-w

**Published:** 2020-05-07

**Authors:** Karin Haar, Aala El-Khani, Virginia Molgaard, Wadih Maalouf, the Afghanistan field implementation team, Mohammad Raza Stanikzai, Mohammad Nader, Leland Molgaard, Rachel Calam, Debborah Allen, Lindsey Coombes, Gilberto Gerra, Giovanna Campello, Nina Fabiola Montero Salas, Hashmatullah Nazir

**Affiliations:** 1 Prevention, Treatment and Rehabilitation Section, Drug Prevention and Health Branch, Division of Operations, United Nations Office on Drugs and Crime (UNODC), Wagramer Strasse 5, A-1400 Vienna, Austria; 2 Department of Human Development and Family Studies Office, 2625 N Loop Dr Ste 500 Research Park 2, Ames, IA 50010 USA

**Keywords:** Humanitarian challenged settings, Afghanistan, Family skills programme, Child mental health, Parenting practices

## Abstract

**Background:**

Children living in challenged humanitarian settings (including those in rural/underserved areas, the displaced, refugees, in conflict/post conflict situations) are at greater risk of mental health difficulties or behavioural problems, with caregivers acting as their main protective factors. While many family skills programmes exist, very few were developed for, or piloted in, low resource settings (settings with limited infrastructure, typical of humanitarian settings). We therefore designed a brief and light programme; the Strong Families (SF) programme, consisting of 5 h contact time over 3 weeks. We conducted a pilot study with the aim to test the feasibility of implementation, and a preliminary look at the effectiveness of SF, in improving child behaviour and family functioning in families living in Afghanistan.

**Methods:**

We recruited female caregivers and children aged 8–12 years through schools and drug treatment centres in Afghanistan and enrolled them in the SF programme. Demographic data, emotional and behavioural difficulties of children and parental skills and family adjustment measures were collected from caregivers before, 2 and 6 weeks after the intervention. Outcome was assessed through the SDQ (Strengths and Difficulties Questionnaire), assessing children’s behavioural, emotional, and social issues, and PAFAS (Parenting and Family Adjustment Scales), measuring parenting practices and family functioning.

**Results:**

We enrolled 72 families in the programme with a 93.1% retention rate (*n* = 67) for data collection 6 weeks post intervention. Mean age of caregivers was 36.1 years, they had 3.8 children on average and 91.7% of them had experienced war/armed conflict in their past. The average total difficulty score of the SDQ (ranging from 0 to 40, with scores above 16 being indicative of high problems) of the 72 children reduced significantly, from 17.8 at pre-test to 12.9 at post-test and 10.6 at second follow-up, with no difference in gender and most noticeably amongst those with the highest scores at baseline. Likewise, PAFAS scores decreased significantly after the programme, again with caregivers with the highest scores at baseline improving most.

**Conclusions:**

The implementation of a brief family skills programme was seemingly effective and feasible in a resource-limited setting and positively improved child mental health and parenting practices and family adjustment skills. These results suggest the value of such a programme and call for further validation through other methods of impact assessment and outcome evaluation.

**Trial registration:**

ISRCTN76509384. Retrospectively registered on March 9, 2020.

## Background

Humanitarian or challenged settings are those threatening in terms of health, safety or well-being of a large group of people, such as communities that have faced natural disasters, conflict and complex political emergencies. These could include refugee, displacement or conflict/ post-conflict settings, rural or underserved areas where the level of stress is elevated. Children living in such humanitarian settings are at greater risk of different vulnerabilities including showing signs of mental health difficulties or behavioural problems [1, 2]. This represents a long-term risk as many mental health problems begin in youth and are related to other poor health and developmental outcomes, such as violence, lower educational achievement and substance abuse [3, 4].

Further to the aforementioned direct impact on children, social inequalities resulting from political conflict such as family instability or security, poor caregiver mental health due to prolonged periods of stress further exacerbate the vulnerability faced by the children. Whereas one of the most important factors preventing psychological morbidity in children affected by armed conflict and compounding challenges may be parental support and monitoring [5–7]. Primary caregivers, play a crucial role in protecting children’s mental health in challenging contexts buffering the children’s mental health outcomes in times of danger, upheaval, and uncertainty [8]. Hence, for such children and families, parental and family factors are even more important in achieving positive outcomes [9].

Family skills programmes offer a combination of parenting knowledge, skill building, competency enhancement and support [10]. They aim to strengthen family protective factors such as communication, trust, problem-solving skills and conflict resolution, and strengthen the bonding and attachment between caregivers and children.

Evidence of the effectiveness of parenting interventions in high income and more stable contexts indicates potential for such programmes in improving caregiver–child relationships, and subsequent child behaviour and emotional wellbeing in conflict-affected and low-resource settings [11, 12].Overall, very few family skills programmes were designed to serve the needs of families living in low resource settings (settings with limited infrastructure, typical of humanitarian settings) [13]. In a recent review of existing evidence of parenting programmes in low and middle income countries [12], only one evaluated intervention incorporated sessions tailored to a conflict-affected population in Northern Uganda [14]. To fill this gap, the United Nations Office on Drugs and Crime (UNODC), with the support of experts in the field, developed the Strong Families programme. The Strong Families programme is a selective evidence-informed prevention intervention programme designed to improve parenting skills, child well-being and family mental health, amongst those with children aged between 8 and 15 years. Namely it was tailored for challenged and humanitarian settings. It was developed ensuring it to be brief (as few sessions as possible), “light” (requiring an infrastructure that is easy to mobilise and train), evidence-informed, suitable for low-resource settings, open source (to allow benefitting counterparts to have national ownership to bring it to scale at minimum cost) and cost-effective.

The vision of Strong Families is to support families in recognising their strengths and skills and to make them stronger by sharing their challenges as well as the things that work for them [15]. It operates through the logic model outlined in Table 1 and was first piloted in Afghanistan [16, 17].

**Table 1:** Logic Model of the Strong Families Programme

**Program components**	**Program process to address underlying causes**	**Short term participant and family impact**	**Long term impact**
**Caregiver sessions** **Goal**: Normalise and manage stress Improve parenting confidence and develop positive parenting strategies Enancing resources to deal with stress **Child sessions** **Goal:** Improve mental health outcomes, better deal with stress, reduce challenging behaviour **Family sessions** **Goal:** Improved communication and relationships	**Decrease risk factors** Favourable attitudes towards coercive parenting strategies; Poor family management skills High levels of stress Environment favouring early initiation of drug use and of conflict and violenc **Increase protective factors** Improved family interaction Enhanced relationships, non-violent discipline, prosocial involvement, caregiver social support	Improved caregiver confidence in family management skills Improved caregiving in parenting skills Improved child behaviour Reduced aggressive and hostile behaviours Increased capacity to cope with stress Improved mental health outcomes in children and parents	Reduction in violence Reduction in substance abuse Reduction in risky behaviours Improved mental health for caregivers and children

Afghanistan is one of the five poorest countries in the world [18], its public health profile indicates a dangerous combination of ongoing conflict and chronic poverty making it a complicated challenged humanitarian context [8]. The international community has put considerable effort into rebuilding Afghanistan, yet the country faces many challenges: only 46% of people have access to safe drinking water and 92% do not have access to adequate sanitation [19]. In addition, while Afghanistan has had a long history of invasion and war, in recent years the country has seen an increase in violence and conflict [20] leaving many Afghans now internally displaced in various parts of the country. Drug use remains a major health and economic problem for Afghans. By March 2014, Afghanistan produced almost three quarters of the world’s illicit opium [21]. While a significant amount was exported, in 2009, almost 10% of Afghans aged between 15 and 64 years were using drugs, approximately twice the global average, with one of the highest opiate prevalence rates in the world [21, 22]. In a household survey in 2010–12, an opioid prevalence of 5.6% was found, which could even have been higher were homeless people added [23].

Afghan women and children in particular, experience high levels of difficulties affecting their mental health [24–26]. One study found 60% of Afghan women scored high on a self-report measure for depression [24]. This is often linked to exposure to past trauma [27] and ongoing social and material stressors [28, 29]. Decades of war and conflict have also had a significant impact on health and well-being across almost all domains of children’s lives, due to exposure to violence, ongoing insecurity, disrupted networks of social support and poor health. One study showed that by 11–16 years of age, Afghan children experience mental health problems that fall within the expected range of psychiatric difficulties and post-traumatic stress in war-affected populations [29]. Growing up in Afghanistan may lead to exposure to multiple forms of violence through childhood and adolescence. The high levels of exposure to war trauma constituted a risk factor for punitive and neglecting parenting, which was then associated with poor child mental health outcomes [30]. This is consistent with other research by Panter-Brick and colleagues with Afghan families, where family-level violence (including family conflicts as well as past year reports of violence such as experiencing and/or witnessing severe beatings) was found to predict negative changes in children’s mental health one year post initial assessment [8]. Significantly, violence negatively impacted the well-being of both children and parents.

The aim of our pilot study was to test the feasibility of implementation and a preliminary look at the effectiveness of the Strong Families programme in improving child behaviour and family functioning in families living within the context of Afghanistan.

## Methods

### Programme intervention

The Strong Families programme [16, 17] is a three sessions (7 components requiring 5-h of invested time by the families in total; Table 2) group intervention attended by children and their primary caregivers over a time span of 3 weeks (one session per week). In week one, a group of 12 caregivers meet for the 1-h caregiver pre-session. In weeks two and three, the same 12 caregivers meet again in one room, and their 12 children meet in parallel in another room for the child sessions. After these 1-h parallel sessions, all caregivers and children immediately meet in one room for another 1-hour for a joint family session. The session of week one (caregiver pre-session) explores parents challenges and develops ways to better deal with stress. In week two, caregivers discuss the means of showing love while at the same time having and enforcing limits and listening to children, while the children learn how to deal with stress. During the family session they practice positive communication and are encouraged to practice stress relief techniques together. In week three, parents learn to encourage good behaviour and discourage misbehaviour, while children explore rules and responsibilities and think about future goals in addition to the important roles their caregivers play in their lives. In the final family session, caregivers and children learn about family values and practice sharing appreciation to each other.

**Table 2:** Structure of Strong Families Program

**Week 1**	**Week 2**		**Week 3**	
**Caregiver pre-session** Understanding Strengths and Stresses	**Caregiver session 1** Using love and limits	*In parallel*	**Caregiver session 2** Teaching children what is right	*In parallel*
	**Child session 1** Learning about stress		**Child session 2** Following rules and appreciating parents	
	**Family session 1** Learning about each other		**Family session 2** Supporting values and dreams	

Cultural adaptation of the Strong Families programme to the Afghan context was assured through a 3-day working group with representatives from the Afghan Ministries of Public Health (MOPH), Labour and Social Affairs, Counter Narcotics (MCN), Education (MOE), Afghan NGOs (non-governmental organisations), the programme developers and UNODC.

The translation of the training material for facilitators and questionnaires for the participants into Dari was performed by a qualified and experienced translation company and reviewed by local researchers.

### Study design and participants

An open, pilot feasibility and acceptability trial was conducted with an embedded effectiveness evaluation including a prospective collection of outcome data assessing changes in children’s behaviour, parenting skills and family adjustment in caregivers.

Participants were selected based on being a female caregiver to a child between the age of 8–12 years. The cultural adaptation team, consisting of Afghan representatives who reviewed the materials, pointed out that religious and cultural norms do not allow for men and women, who are not related, to mix together. Respecting the cultural context, and given the stage and intent of the current pilot, it was decided to target either males or females caregivers at this time. As females are most often the primary caregivers in Afghan families, female caregivers and their children (of either gender) were chosen.

Sampling was opportunistic, using a ‘universal’ approach, in which facilitators recruited families from the general population, not targeting specifically those with a particular risk. Inclusion criteria included speaking Dari, willing to take part in the programme and being in the town for the duration of the whole study and measurement meetings. Families that had already taken part in another family skills training programme in the past 6 months or where the caregiver lived separately from the child were excluded from the programme.

### Procedure

**Fig. 1:**
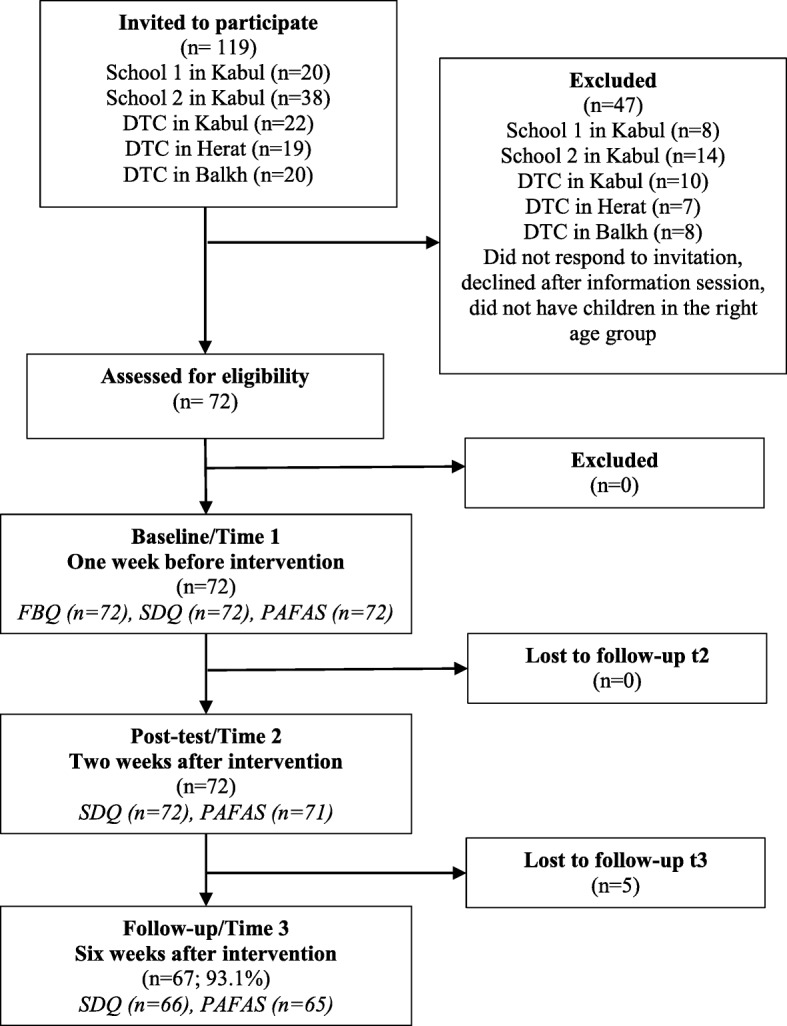
Recruitment of participants, follow-up and missing data from SDQ and PAFAS over time

The pilot study took place in three towns in Afghanistan, namely in Kabul, in Mazār-i-Sharīf (Balkh) and in Herat between July and October 2018. Based on UNODC’s mandate, and under the umbrella of the National Drug Control Strategy, UNODC approached MCN and MOPH to facilitate the implementation of the programme in Afghanistan who later expanded the collaboration with MOE, the Ministry of Women Affairs and the Ministry of Health. As displayed in Fig. 1, families were recruited via two high schools and one 100 beds Drug Treatment Centre (DTC) for women and children in Kabul, a 150 beds drug demand reduction (DDR) hospital in Balkh and a women DTC in Herat.

The schools were selected by the MOE based on the main criteria of having easy access of the families to the school and the provision of two class rooms for the programme to run. Caregiver information sheets were distributed to all children aged eight to 12, who attended the participating schools, to take home to their caregivers. Female caregivers were invited via a self-referral process to attend an information session where they were given further verbal and written information and questions were answered. Caregivers were asked to phone the school within the next 4 days if they wanted to participate. The first to call the school were invited to take part in the pilot study and attended a baseline measurement session the following week in which written informed consent was obtained prior to data collection.

In Afghanistan, DTC provide health education and services to the most affected families, are well connected with local communities and most families have access to them. The women DTC have four components of service delivery: home-based, outreach, outpatient and residential. The home-based part raises awareness and provides education and information to surrounding affected families whereas the outreach component raises awareness and provides screening and brief intervention in the communities, particularly focussing on the identification of the vulnerable and at-risk women and children and providing information and education program at home. The outpatient and residential parts of the DTCs are for drug treatment and rehabilitation of women suffering from drug use disorders. Further, the existing Afghan drug demand reduction policy and strategy recommends integration of the drug prevention in health services. DTC were included so as not to bias against families in which children were out of school. Again, female caregivers from the home-based or outreach component of DTC were given a caregiver information sheet and invited via the same self-referral process to attend the information session at a participating school, and the same recruitment process took place. Noting that the recruitment strategy was the same in both centres, all interested families were invited. The DTC were implicated given their interest to be involved in prevention responses. In general, we did not formally assess clinical diagnoses in caregivers or their children, however at the first session, caregivers were handed out leaflets with information as to where to get help in case they observe more severe stress reactions, severe trauma or other physical, mental or sexual health issues in themselves or their children.

Seventeen facilitators were selected through schools and health settings (13 females and four males), local NGOs, MCN and MOPH. Their backgrounds were mixed, some teachers, some caregivers who had previously taken part in family skills programmes, psychologists and social workers. This mix in backgrounds was important as the programme is designed to be run by lay people without a particular expertise. Facilitators took part in a 3-day training programme in New Delhi-India, delivered by the developers of the programme who are experienced international trainers. Clarification of questions and more practising of difficult parts was assured via a national refresher training prior to roll out to families, as well as through remote monitoring via weekly Skype calls between the developers from the UK, UNODC staff in Vienna and coordinators in Kabul to ensure coverage of all field questions and adherence to protocol.

### Confidentiality and ethical considerations

This pilot study has been thoroughly reviewed and approved by the UNODC Drug Prevention and Health Branch in the Headquarters office of Vienna and the national field office in Kabul as well as the associated national ministries (Afghan Ministry of Counter Narcotics, Ministry of Public Health, Ministry of Labour and Social Affairs, Ministry of Education, Ministry of Women Affairs and Ministry of Health) and NGOs that supported the programme as alternative to ethics committee review. The Strong Families programme has been thoroughly analysed and, after approval, has been integrated in the National Drug Demand Reduction policy 2019–2023 for Afghanistan, as well as the National Drug Demand Reduction Strategy 2018–2022. Further, the donor to the programme development and implementation, US-INL (Bureau of International Narcotics and Law Enforcement Affairs) under the U.S. Department of State, had reviewed and approved the proposal before initiating the trial. The piloting was performed in accordance with the ethical standards of the 1964 Helsinki declaration and its later amendments or comparable ethical standards.

The Chief Investigator and the research team assured the confidentiality of participants in accordance with the Data Protection Act 1998. Each participant was assigned a unique identification number to ensure matching of all questionnaires. All data collected as part of the trial were treated as confidential and were only be viewed by members of the trial team; anonymised data were used wherever possible.

All caregivers completed and signed a consent form at the first evaluation meeting, as all children were under the age of 16 years, they consented as a legal guardian for their children. No measures were taken directly from children, the reported results are from caregiver measure reports, thus children were not required to complete an assent form. In addition to written information being provided in the form of the Participant Information Sheets for the caregiver, participants were provided with a verbal explanation of the evaluation method at the first meeting and again when they attended the first data collection session.

### Data collection

Data on demographics, emotional and behavioural difficulties of children and parental skills and family adjustment measures were collected from caregivers through self-administered questionnaires.

Two outcome measures were completed by participants at baseline (i.e. 1 week before intervention delivery) (t1) and 2 weeks (t2) and 6 weeks (t3) after intervention delivery. These were the paper-based Strengths and Difficulties Questionnaire (SDQ) and a Parenting and Family Adjustment Scales (PAFAS). A standardized Family Background Questionnaire (FBQ) was completed at t1 to collect demographic characteristics.

The SDQ is a commonly used short screening tool to assess children’s behavioural, emotional, and social issues over the last six months. It is available in over 40 different languages and is frequently used for research purposes to examine children’s mental well-being. The advantages of the SDQ were its compact format (relative to the previously long-established and highly respected Rutter behavioural screening questionnaires) in covering the strengths as well as difficulties in inattention, peer relationships, and prosocial behavior [31]. It has shown good psychometric properties and has been used in Afghanistan previously [32]. The SDQ examines 25 attributes, each rated on a 3-point scale ranging from 0 (“Not True”) to 2 (“Certainly True”). The answers can be summed into five subscales (ranging from 0 to 10 points each), including items such as emotional symptoms (e.g. “Often unhappy”) and conduct problems (e.g. “Often fights with other children”). The Total Difficulties score (ranging from 0 to 40 points, with higher scores indicating higher levels of difficulties) is calculated from four of the subscales excluding prosocial behaviours [31]. We used the cut-points for the 4-banded categorization of the SDQ scores to classify continuous measures into “close to average”, “slightly raised/lowered”, “high/low” and “very high/low” risk [33].

The PAFAS is a brief 30-item, user-friendly questionnaire measuring parenting practices and family functioning, which are known risk or protective factors for child emotional or behavioural problems. PAFAS was developed to assess changes in parenting skills and family relationships before and after public health and individual or group parenting interventions. It comprises two scales: (i) Parenting, measuring parenting practices (e.g., descriptive praise, logical consequences) and the quality or parent-child relationship (e.g. level of reciprocal warmth, parental satisfaction with the relationship to the child) and (ii) Family Adjustment, measuring parental emotional adjustment (e.g. level of stress, depression and anxiety experienced by a parent in his or her role) and positive family relationships (e.g. supportive and conflict-free family environment) and parental teamwork (e.g. social support received from the partner in the parenting role) [34]. The PAFAS subscales have shown good internal consistencies in two Australian samples (ranging from .70 to .87) and satisfactory construct and predictive validity [34]. Furthermore, the measure has been validated in other cultures, such as Panama [35] and China [36]. In both studies, the factor structures in the original PAFAS measure were mostly retained with fewer items and adequate internal consistencies (ranging from .50 to .82 and from .65 to .95 for Chinese and Panamanian parents, respectively). PAFAS has been recently used with Arabic speaking families living in political conflict in the West bank [37]. To our knowledge there are no clinically relevant cut-off points available. For sub-analyses purposes, a cut off at the 75th percentile was assumed Participants with scores above the upper quartile (Q3) at baseline, 25% of participants, represent families with higher levels of difficulties.

The implementation process was evaluated based on the methods and fidelity assessment sheets previously described by Segrott et al. [38] In addition we provided questionnaires to be filled in by observers. Basic data on the number of sessions were provided from the coordinator in the field. In each session of the Strong Families programme, an independent observer assessed predefined indicators, and facilitators were asked to complete another questionnaire. Indicators to measure different process evaluation components were modified from the ones used for the SFP 10–14 in the UK [38] to match the Strong Families programme, as indicated in Table 6.

### Statistical analysis

The data was entered in Epidata version 3.1 and analysed using GNU PSPP Statistical Analysis Software, version 1.0.1 and IBM SPSS Statistics version 25. Prior to analysis, data completeness and plausibility checking was assured.

Continuous variables are presented as mean, standard deviation (SD) and minimum and maximum scores. Categorical data was summarized by frequencies and proportions.

A visual inspection of the histograms, Q-Q plots and box plots as well as the calculation of the skewness and kurtosis z-values within a range of +/− 1.96 showed that data were approximately normally distributed.

In the event of normality, to compare means in demographic characteristics, a 2-sample t-test was used for continuous variables, while a chi-square test for categorical data.

For comparison of means or ranks at the different time points (pre-test, post-test and follow-up), a repeated measures ANOVA was used for normally distributed data with post-hoc tests using Bonferroni corrections. In case Mauchly’s Test of Sphericity indicated that the assumption of sphericity had been violated, a Greenhouse-Geisser correction was used. A Friedman’s ANOVA with a Wilcoxon’s Signed Rank test as post-hoc test were used as non-parametric tests.

For potential gender-based effects and to compare SDQ scores at each timepoint between boys and girls we used a 2-sample t-test for normally distributed data or a Mann-Whitney-U test if data were not assumed to be normally distributed. Participants with very high (20–40 points) scores at baseline on the total difficulty scale of the SDQ were analysed separately for each of the subscales to show potential effects on all subscales in people with most difficulties at start point.

Similarly, a sub-analysis on families cut off at the 75th percentile in each subcategory of the PAFAS at baseline was performed to compare the effects on those families with high problems at baseline to those with less difficulties. Statistical significance level was set at *p*-value lower than 0.05.

For process evaluation data, proportions were calculated out of all data obtained from facilitators and observers.

## Results

### Recruitment, follow- up and missing data

Overall, 72 families were enrolled in the programme (Fig. 1), 48 in Kabul, 12 in Balkh and 12 in Herat. The five caregivers who were lost to follow up at t3 were significantly younger (mean age 28.2 compared to 36.7 years; t_69_ = − 2.44; *p* = 0.017). No other difference in recruitment site, marital status, education or mean number of children was found.

Missing data on individual questions within the FBQ were regarded as minor and non-systematic. Individual PAFAS answers were missing in less than 5% of cases, apart from PAFAS question four (“I threaten something when my child misbehaves”) at time two (7/71 missing), for PAFAS question 15 (“I enjoy giving my child hugs and kisses”), where eight, 12 and 12 answers were missing at the 3 time-points. Not surprisingly, PAFAS questions 28–30 regarding parental teamwork (“I work as a team/disagree/ have a good relationship with my partner”) were mainly answered by persons in a relationship. No question in the SDQ at any timepoint was answered by less than 5% of participants. No imputation of the data was performed, participants with missing data were excluded from the respective analyses.

### Demographics of study participants

On average the caregivers were 36 years old, with an age range between 17 and 50 years. There was no difference in caregiver’s age between the three geographical sites. All 72 caregivers were female, 66% were married, 41% had primary school or less and 25% were working full time, as shown in Table 3. There was a significant difference in marital status and education between caregivers recruited through DTCs and high schools, but there were none regarding their partner’s education or their or their partner’s work status. Children from parents recruited through DTC were significantly older than those recruited through high schools and all caregivers recruited through DTC had experienced war or armed conflict, compared to 83% of those recruited through high schools (Table 3). All 72 participants identified most strongly with the Afghan ethnic or cultural group and reported Afghanistan as their country of origin.

**Table 3:** Demographic characteristics of study participants in Afghanistan (*n* = 72)

		**Total**	**DTC**	**High school**	***p*-value**	**Chi**^**2**^, **t-test**
		Mean (SD); n (%)	Mean (SD); n (%)	Mean (SD); n (%)		
**Caregiver demographics**						
**Age (in years)**		36.1 (7.78)	36.4 (6.45)	35.8 (8.97)	0.773	t_69_ = −0.29
**Marital status**	Married	46 (65.7%)	20 (57.1%)	26 (74.3%)	*0.006*	Χ^2^ = 14.60
	Divorced	2 (2.9%)	2 (5.7%)	0		
	Single	5 (7.1%)	0	5 (14.3%)		
	Cohabiting	6 (8.6%)	6 (17.1%)	0		
	Widow	11 (15.7%)	7 (20.0%)	4 (11.4%)		
**Education**	Primary school or less	29 (41.4%)	20 (57%)	9 (26%)	*0.013*	Χ^2^ = 12.76
	Some high school	11 (15.7%)	7 (20%)	4 (11%)		
	Completed high school	10 (14.3%)	4 (11%)	6 (17%)		
	University degree	19 (27.1%)	4 (11%)	15 (43%)		
	Post-graduate	1 (1.4%)	0	1 (3%)		
**Partner’s education**	Primary school or less	22 (37.3%)	14 (50%)	8 (25.8%)	0.180	Χ^2^ = 7.60
	Some high school	4 (6.8%)	3 (10.7%)	1 (3.2%)		
	Completed high school	12 (20.3%)	5 (17.9%)	7 (22.6%)		
	University degree	17 (28.8%)	4 (14.3%)	13 (41.9%)		
	Post-graduate	2 (3.4%)	1 (3.6%)	1 (3.2%)		
**Work status**	Full time	17 (25.4%)	11 (32%)	6 (18%)	0.053	Χ^2^ = 9.36
	Part time	7 (10.5%)	3 (9%)	4 (12%)		
	Not working but looking for a job	17 (25.4%)	4 (12%)	13 (39%)		
	Home based paid work	9 (13.4%)	4 (12%)	5 (15%)		
	Not working	17 (25.4%)	12 (35%)	5 (15%)		
**Partner’s work status**	Full time	32 (59.3%)	16 (61.5%)	16 (57.1%)	0.857	Χ^2^ = 1.32
	Part time	7 (13%)	2 (7.7%)	5 (17.9%)		
	Not working but looking for a job	4 (7.4%)	2 (7.7%)	2 (7.1%)		
	Home based paid work	2 (3.7%)	1 (3.9%)	1 (3.6%)		
	Not working	9 (16.7%)	5 (19.2%)	4 (14.3%)		
**Number of children**		3.8 (1.9)	3.94 (1.81)	3.65 (2.01)	0.529	t_64_ = −0.63
**Experienced war or armed conflict**	Yes	66 (91.7%)	36 100%)	30 (83.3%)	*0.011*	Χ^2^ = 6.55
	No	6 (8.3%)	0	6 (16.7%)		
**Child demographics**						
**Age of child taking part in the programme (in years)**		9.6 (2.05)	10.19 (1.89)	8.97 (2.04)	*0.011*	t_69_ = −2.62
**Gender of child in the programme**	Male	38 (53.5%)	19 (52.8%)	19 (54.3%)	0.899	Χ^2^ = 0.02
	Female	33 (46.5%)	17 (47.2%)	16 (45.7%)		

### Child behaviour, as assessed through the SDQ

Overall, the total difficulty score of the SDQ reduced significantly over time, from 17.8 at pre-test to 12.9 at post-test and 10.6 at follow-up (*p* < 0.001). Likewise, all SDQ subscales declined over time, as shown in Table 4.

**Table 4:** Mean SDQ score over time overall, by gender and in participants with very high total scores at baseline

**Gender-based analysis**		**Pre-test** Mean (SD) [Min-Max]	**Post-test** Mean (SD) [Min-Max]	**Follow-up** Mean (SD) [Min-Max]	***ANOVA***		***Pairwise comparison***
					***F (df***_***time***_***, df***_***error***_***); χ***^***2***^***(df)***	***p-value***	
SDQ subscales							
**Emotional problem scale** **[0–10]**	Boys (*n* = 38)	5.50 (2.05) [1–10]	4.29 (2.02) [0–9]	3.46 (2.19) [0–8]	F(2,31) = 18.03	*p < 0.001*	^b, d^
	Girls (*n* = 33)	4.91 (1.96) [1–8]	4.0 (1.97) [0–9]	2.70 (1.56) [0–6]	F(2,24) = 15.02	*p < 0.001*	^c, d^
	**Overall**	**5.24** **(2.0)** **[1–10]**	**4.15** **(1.97)** **[0–9]**	**3.14** **(1.95)** **[0–8]**	**F(2,58) = 28.90**	***p < 0.001***	^b, c, d^
**Conduct problem scale** **[0–10]**	Boys (*n* = 38)	3.77 (1.83) [0–7]	2.50 (1.31) [0–6]	2.11 (1.63) [0–7]	F(2,29) = 4.69	*p = 0.017*	^b,d^
	Girls (*n* = 33)	3.30 (2.07) [0–7]	1.56^a^ (1.46) [0–6]	1.64 (1.28) [0–4]	χ^2^(2) = 15.31	*p < 0.001*	^b, d^
	**Overall**	**3.55** **(1.94)** **[0–7]**	**2.03**^**a**^ **(1.45)** **[0–6]**	**1.91**^**a**^ **(1.43)** **[0–7]**	**χ**^**2**^**(2) = 25.40**	***p < 0.001***	^b, d^
**Hyperactivity scale** **[0–10]**	Boys (*n* = 38)	5.42 (2.02) [1–10]	3.62 (1.86) [0–8]	3.19 (2.04) [0–9]	F(2,33) = 17.55	*p < 0.001*	^b, d^
	Girls (*n* = 33)	4.94^1^ (2.20) [0–9]	3.61 (1.95) [0–8]	2.86 (1.74) [0–6]	χ^2^(2) = 12.65	*p* = 0.002	^b, d^
	**Overall**	**5.21** **(2.09)** **[0–10]**	**3.59** **(1.89)** **[0–8]**	**3.08** **(1.91)** **[0–9]**	**F(2,61) = 24.20**	***p < 0.001***	^b, d^
**Peer problem scale** **[0–10]**	Boys (n = 38)	3.80 (1.47) [0–7]	3.14 (1.59) [0–7]	2.91 (1.50) [0–6]	F(2,30) = 4.95	*p = 0.014*	^d^
	Girls (*n* = 33)	3.75 (1.57) [0–6]	3.0 (1.32) [0–5]	2.61 (1.26) [0–5]	F(2,24) = 2.43	*p* = 0.11	
	**Overall**	**3.78** **(1.51)** **[0–7]**	**3.07** **(1.45)** **[0–7]**	**2.78** **(1.39)** **[0–6]**	**F(2,56) = 7.34**	***p = 0.001***	^d^
**Prosocial scale** **[10–0]**	Boys (*n* = 38)	6.33 (1.95) [2–10]	7.74 (1.40) [4–10]	7.68 (1.96) [3–10]	F(2,29) = 8.71	*p = 0.001*	^b, d^
	Girls (*n* = 33)	6.91 (1.93) [4–10]	8.13 (1.45) [5–10]	8.04 (1.53) [5–10]	F(2,24) = 6.82	*p = 0.005*	^b, d^
	**Overall**	**6.61** **(1.93)** **[2–10]**	**7.90** **(1.44)** **[4–10]**	**7.80** **(1.79)** **[3–10]**	**F(2,56) = 15.94**	***p < 0.001***	^b, d^
**Total Difficulty Scale** **[0–40]**	Boys (*n* = 38)	18.52 (5.08) [10–32]	13.61 (4.53) [6–22]	11.24 (5.56) [2–23]	F(2,25) = 22.14	*p < 0.001*	^b, d^
	Girls (*n* = 33)	16.97 (6.23) [4–26]	12.27 (5.62) [3–27]	9.74 (4.17) [2–19]	F(2,21) = 15.69	*p < 0.001*	^d^
	**Overall**	**17.77** **(5.67)** **[4–32]**	**12.9** **(5.07)** **[3–27]**	**10.62**^**a**^**(4.98)** **[2–23]**	**χ**^**2**^**(2) = 34.16**	***p < 0.001***	^b, c, d^
**Very high (20+) on total difficulty scale (n = 24; 12 boys, 12 girls) at baseline**							
Emotional problem scale		6.96 (1.33) [4–10]	4.46 (2.21) [1–9]	3.81 (2.29) [0–8]	F(2,19) = 24.99	*p < 0.001*	^b, d^
Conduct problem scale		4.79 (1.50) [2–7]	2.26 (1.81) [0–6]	1.86 (1.35) [0–5]	F(2,18) = 18.66	*p < 0.001*	^b, d^
Hyperactivity scale		7.17 (1.20) [5–10]	3.96 (1.94) [1–8]	3.38 (2.22) [0–9]	F(2,19) = 27.55	*p < 0.001*	^b, d^
Peer problem scale		4.46 (1.32) [2–6]	2.78 (1.70) [0–6]	2.75 (1.48) [0–6]	F(2,17) = 6.43	*p = 0.008*	^b, d^
Prosocial scale		6.26 (2.30) [2–10]	8.22 (1.54) [4–10]	7.57 (2.20) [3–10]	F(2,17) = 15.53	*p < 0.001*	^b, d^
Total Difficulty Scale		23.38^a^ (2.95) [20–32]	13.91 (5.93) [3–27]	11.55 (5.64) [4–23]	χ^2^(2) = 21.33	*p < 0.001*	^b, d^

*statistically significant (p < 0.05)*; SD: standard deviation, ^a^ Data not normally distributed, non-parametrical tests used for all statistics involving this group; ^b^ significant difference between t1 and t2, ^c^ significant difference between t2 and t3, ^d^ significant difference between t1 and t3

Scores for both, boys and girls declined significantly, and no difference in gender could be found, apart from the conduct problem scale, where a significant difference at time 2 (*p* = 0.008), that dissipated at time 3.

There was no difference in SDQ scores at any time between participants recruited through DTCs or high schools. In children with very high (20+) scores on the total difficulty scale (*n* = 24; 12 boys, 12 girls) at baseline, highly significant reductions in all sub-scores as well as the total difficulty score could be found after the programme. There was no change between time 2 and time 3, however, overall, the effects were long-lasting, with highly significant differences at time 3 compared to baseline (Table 4).

### Parenting practices and parent and family adjustment, as assessed through the PAFAS

Overall, parenting practice scores decreased significantly as assessed through all four PAFAS subscales between baseline and post-intervention and at follow-up compared to baseline. Across all parenting practices subscales, participants with scores above the 75 percentile at baseline had their scores more significantly reduced after the intervention compared to the rest of the sample (Table 5).

**Table 5:** Mean PAFAS scores over time overall and for families above and below the 75th percentile in each subcategory at baseline

**PAFAS**	**Pre-test** Mean (SD) [Min-Max]	**Post-test **Mean (SD) [Min-Max]	**Follow-up** Mean (SD) [Min-Max]	***ANOVA***		***Pairwise comparison***
				***F (df***_***time***_***, df***_***error***_***); χ***^***2***^***(df)***	***p-value***	
PARENTING						
**Parental Consistency** [0–15]						
≤ 75th percentile Score 0–8 (*n* = 41)	6.39^a^ (1.51) [1–8]	5.89 (2.67) [1–12]	6.60 (2.13) [3–11]	χ^2^(2)= 3.49	*p* = 0.175	
>75th percentile Score 9+ (*n* = 24)	10.13 (1.08) [9–12]	6.90 (2.22) [4–11]	7.15 (1.84) [4–10]	F(2,15) = 29.18	*p < 0.001*	b, d
**Overall**	**7.77** **(2.27)** **[1–12]**	**6.28** **(2.46)** **[1–12]**	**6.90** **(2.02)** **[3–11]**	**F(2,45)=** **7.72**	***p = 0.001***	b, d
**Coercive Parenting** [0–15]						
≤ 75th percentile Score 0–10 (*n* = 47)	6.51 (2.23) [1–10]	5.36 (3.06) [0–11]	4.79 (2.97) [0–12]	F(2.36)= 4.83	*p = 0.014*	b, d
>75th percentile Score 11+ (*n* = 22)	12.73 (1.28) [11–15]	5.00 (2.65) [1–11]	4.76 (2.83) [0–9]	F(2.19)= 96.06	*p < 0.001*	b, d
**Overall**	**8.49** **(3.52)** **[1–15]**	**5.23** **(2.88)** **[0–11]**	**4.85** **(2.84)** **[0–12]**	**F(1.49,86.76)=** **37.72**	***p < 0.001***	b, d
**Positive Encouragement** [0–9]						
≤ 75th percentile Score 0–1 (*n* = 37)	0.62^a^ (0.49) [0–1]	0.83^a^ (1.12) [0–5]	0.63^a^ (0.96) [0–3]	χ^2^(2)= 0.46	*p* = 0.796	
>75th percentile Score 2+ (*n* = 33)	3.18^a^ (1.47) [2–7]	0.80^a^ (0.96) [0–3]	0.90 (0.84) [0–3]	χ^2^(2)= 31.76	*p < 0.001*	b, d
**Overall**	**1.83**^**a**^ **(1.67)** **[0–7]**	**0.82**^**a**^ **(1.04)** **[0–5]**	**0.77**^**a**^ **(0.89)** **[0–3]**	**χ**^**2**^**(2)=** **14.83**	***p = 0.001***	b, d
**Parent-child Relationship** [0–15]						
≤ 75th percentile Score 0–2 (*n* = 44)	0.73 (0.69) [0–2]	0.66^a^ (1.15) [0–5]	0.31^a^ (0.80) [0–3]	χ^2^(2)= 9.27	*p = 0.010*	d
>75th percentile Score 3+ (*n* = 20)	4.90^a^ (2.43) [3–11]	0.93^a^ (1.21) [0–4]	0.50^a^ (0.94) [0–3]	χ^2^(2)= 17.89	*p < 0.001*	b, d
**Overall**	**2.03**^**a**^ **(2.43)** **[0–11]**	**0.74**^**a**^ **(1.13)** **[0–5]**	**0.36**^**a**^ **(0.81)** **[0–3]**	**χ**^**2**^**(2)=** **22.54**	***p < 0.001***	b, c, d
FAMILY ADJUSTMENT						
**Parental Adjustment** [0–15]						
≤ 75th percentile Score 0–7 (*n* = 41)	5.12^a^ (1.86) [0–7]	4.30 (1.82) [1–9]	3.94 (2.38) [0–9]	χ^2^(2)= 6.0	*p* = 0.050	
>75th percentile Score 8+ (*n* = 26)	8.73^a^ (1.08) [8–12]	5.14 (2.57) [1–11]	4.81 (2.32) [0–8]	χ^2^(2)= 24.42	*p < 0.001*	b, d
**Overall**	**6.52** **(2.38)** **[0–12]**	**4.57** **(2.18)** **[0–11]**	**4.30** **(2.33)** **[0–9]**	**F(2,50)=** **13.51**	***p < 0.001***	b, d
**Family relationships** [0–12]						
≤ 75th percentile Score 0–4 (*n* = 37)	2.73 (1.41) [0–4]	2.34 (1.98) [0–9]	1.70^a^ (2.04) [0–8]	χ^2^(2)= 7.91	*p = 0.019*	d
>75th percentile Score 5+ (*n* = 33)	6.09^a^ (1.42) [5–9]	2.77 (1.71) [0–7]	2.03^a^ (2.18) [0–8]	χ^2^(2)= 39.94	*p < 0.001*	b, c, d
**Overall**	**4.31** **(2.20)** **[0–9]**	**2.56**^**a**^ **(1.83)** **[0–9]**	**1.89**^**a**^ **(2.09)** **[0–8]**	**χ**^**2**^**(2)=** **40.73**	***p < 0.001***	b. c. d
**Parental teamwork** [0–9]						
≤ 75th percentile Score 0–4 (*n* = 44)	1.95^a^ (1.51) [0–4]	1.67^a^ (1.88) [0–9]	1.28^a^ (1.47) [0–6]	χ^2^(2)= 10.15	*p = 0.006*	d
>75th percentile Score 5+ (*n* = 15)	5.53^a^ (1.06) [5–9]	2.27 (2.09) [0–6]	2.11^a^ (2.47) [0–8]	χ^2^(2)= 8.31	*p = 0.016*	b, d
**Overall**	**2.86** **(2.10)** **[0–9]**	**1.82**^**a**^ **(1.88)** **[0–9]**	**1.38**^**a**^ **(1.71)** **[0–8]**	**χ**^**2**^**(2)=** **17.06**	***p < 0.001***	b, c, d

*statistically significant (p < 0.05)*, *SD* standard deviation, ^a^Data not normally distributed, non-parametrical tests used for all statistics involving this group; ^b^ significant difference between t1 and t2, ^c^ significant difference between t2 and t3, ^d^ significant difference between t1 and t3

Likewise, family adjustment scores improved significantly after the programme, as seen in all three subscales. Again, those with the highest scores at baseline had their scores decline most after the intervention.

### Process evaluation

All process evaluation components and the respective performance indicators showed positive results ≥85%. Assessing fidelity, as previously described [38], overall 88–99% of our activities in all the sessions of the Strong Families programme were covered, with high consistency in facilitators attendance as well as group size. Facilitators reported very high doses received, measured through the interest of children, as well as their caregivers, being 100% in all sessions respectively. Assessing the reach of the programme, overall, 99% of families attended all three sessions of the programme. Inputs, such as the quality of childcare, travel arrangements, refreshments, rooms, materials and equipment were rated positively between 85 and 93% overall, as shown in Table 6.

**Table 6:** Quantitative data sources used to assess implementation of the Strong Families programme, process evaluation components, indicators [38] and performance results

Process evaluation component	Data source	Indicator	Caregiver			Child		Family		Total
			Pre-session	Session 1	Session 2	Session 1	Session 2	Session 1	Session 2	
Dose delivered	Coordinator	Number of sessions delivered	6	6	6	6	6	6	6	**6**
Fidelity	Observer	Percentage of activities reported as covered	91%	88%	77%	93%	93%	83%	93%	**88%**
	Facilitator	Percentage of activities reported as fully/mostly covered as 3/4 (on scale of 1 [not/hardly] to 4 [fully])	100%	100%	100%	94%	100%	100%	100%	**99%**
	Observer	Percentage of programmes with ≥2 facilitators at every session	100%	100%	100%	100%	100%	100%	100%	**100%**
	Observer	Percentage of programmes with ≥1 of the same facilitators at every session	100%			100%		100%		**100%**
	Observer	Percentage of programmes with > 4 and < 13 families ^a^	100%^a^	100%^a^	100%^a^	100%^a^	100%^a^	100%^a^	100%^a^	**100%**^a^
Dose received	Facilitator	Percentage of activities reporting interest of: young people; and parents/carers as 3/4 (on scale of 1 [low] to 4 [high])	100%	100%	100%	100%	100%	100%	100%	**100%**
Reach	Observer	Percentage of families attending all 3 sessions	99%			99%		98%		**99%**
Inputs	Observer	Percentage of sheets with good or very good evaluation of quality of childcare and travel arrangements	100%	100%	100%	100%	50%	100%	100%	**93%**
	Observer	Percentage of sheets with positive evaluation of (area of) refreshments	93%	100%	83%	83%	100%	88%	100%	**92%**
	Observer	Percentage of sheets with positive evaluation of room/materials/equipment	85%	100%	67%	100%	60%	100%	80%	**85%**

^a^ all session actually had between 11 and 12 families as instructed

## Discussion

The Strong Families programme seems feasible to implement in a resource-limited setting, training of facilitators, recruitment, implementation and retention in the programme was high and feasible from first implementation. Moreover, despite being a light intervention, the programme reflected a positive short-term change in scores on the child mental health indicators, as well as the parenting practices and parent and family adjustment skills. Interestingly, the intervention improved the scores on subscales for girls and boys alike, this is important considering the value the United Nations is placing on gender sensitive responses.

Given that the intention of the programme is to support the most vulnerable, it was reassuring to see it did so, and across the different subscales of the SDQ, while being applied universally. This, combined with its lightness, gives the programme a level of flexibility of application avoiding the need for a specific filtering infrastructure that is often hard to find or mobilise in challenged settings.

The Strong Families programme has a disclosing filtering note stating that “it is not meant for adults or children with severe reactions to hardship and stress”. Nevertheless, 92% of caregivers responding to our open invitation in the recruitment area had experienced war or armed conflict, hence elevated scores at baseline were to be expected. As previously described however, war exposure accounted for only about 15% of the variance in PTSD (Post-traumatic stress disorder) symptom levels in Kabul [28, 39], whereas locally salient daily stressors such as overcrowded housing, poverty, unemployment, the security situation, violence in the home, poor health, air pollution, and traffic congestion were better at predicting depression, functional impairment, and general distress in men and women [28]. An overall mean SDQ Total Difficulty score of 17.8 in our pilot study reflects therefore the challenging situations our young study participants were living in.

Interestingly the Strong Family programme seems to strongly benefit children with higher levels of difficulty, per their caregiver’s assessment at baseline, including those with an SDQ score over the cut off score pointing to potential clinically apparent disorders. Although we did not compare the scores to a control group without the intervention, we found it very reassuring that a short and light intervention, such as the Strong Families programme seemed to reduce these scores even after 6 weeks post implementation. This echoes our previous findings of a very light touch intervention consisting of a two-hour parenting seminar and take-home booklet on “caregiving in conflict and post-conflict setting” piloted in Nablus in the State of Palestine [37]. This was further, to the positively tested value of a self-read leaflet on “caregiving in conflict and post-conflict settings” with positive beneficial effects as assessed by families [40]. It is clear, families living in such settings need and can benefit significantly from such family interventions, however the interactive nature of the Strong Families intervention implicating all family members, as compared to the parenting seminar and the leaflet previously described, shows an expected added advantage of building skills and embracement of change within the family. However, the circumstances in such challenged and humanitarian settings sometimes determines what kind of parenting intervention can be disseminated.

The long-term impact of the Strong Families programme, as outlined in the logic model, still needs to be assessed in the future, however, the promising short-term results such as “Improved child behavior”, “Reduced aggressive and hostile behaviors” and “Increased capacity to cope with stress” already seem to positively affect the scores on subscales related to the mental health of the study participants in our pilot study participants.

With the PAFAS we aimed to assess the improvement of parent and family functioning skills, that are known to be protective or risk factors for child emotional and behavioural problems. We could not find any cutoff points that can predict future risk behavior or that would require clinical interventions. Therefore, for our sub-analysis, we set the cutoff at the 75th percentile for each of the domains. Again, those caregivers with higher scores at baseline in the domain of parenting practices, quality of parent–child relationship, parental emotional adjustment, positive family relationships and parental teamwork decreased significantly in scores after the programme. We conclude therefore that the Strong Families programme seems to positively target these areas of promoting child’s positive and prosocial behaviour (e.g. praise through “Using love and limits”), reciprocal warmth and understanding for each other, support to parents to deal with stress and anxiety, setting rules and targets to support a conflict-free family environment and for caregivers to support each other [34]. In our pilot study, we had only few missing data in the answers to the PAFAS questionnaire, apart from question 15 (“I enjoy giving my child hugs, kisses and cuddles”), which had more missing answers at all three timepoints compared to all other questions. We assume that the translation and/or cultural interpretation might have been misunderstood and hence this question was omitted by some participants. A re-evaluation of this question is planned for our upcoming clinical trial. The effect of the Strong Families programme on the domain “Parent-child relationship” (including question 15) might therefore have even been underestimated, as we excluded persons with missing answers from the analyses.

The Strong Families programme was successfully delivered by lay persons in Afghanistan, the fact that these lay facilitators, trained in 3 days to deliver this 5-h programme, showing promisingly positive improvement in the score of children including those reaching clinically significant scores on SDQ symptoms is further encouraging. However, we did not formally assess our study population, and therefore cannot comment on possible clinical diagnoses of our participants at baseline and also, data need to be verified on a larger scale.

It is valuable to note that our pilot study design implicated three different geographical locations within Afghanistan. These sites were selected by the UNODC office in Afghanistan after conducting local assessment for suitability, based on the main criteria of representing the wide variety of ethnic groups in Afghanistan, subcultural differences, geographical location and the availability of two rooms for the programme to run. While this distribution will have increased the external validity of the pilot, the results will require further exploration through a larger and more targeted study to be able to cross-compare results across different sites in support of the potential preparation for a scale up of the implementation. In preparation for such more elaborate plan of analysis, and in interim, further and more targeted research and trials inspired and refined by the observed results are planned.

By implementing the Strong Families programme we aimed to fill a much needed gap through an intervention that focuses at strengthening community and family support for young children, as recommended by Jordans et al. [39] Parent training makes up a highly promising intervention in behavioural disorders of children [41] as well as in the prevention of a variety of negative social outcomes [42].

Despite the promising results of our pilot study, there are also some limitations to it. First, due to cultural/religious reasons, only mothers and children received the programme, and we therefore cannot assess the impact and role of fathers in the Afghan context. Based on personal communication though, fathers would be interested in taking the programme, and the impact on the training of the primary caregiver of a family is one of our objectives of the next phase, a targeted and powered trial based on the refined larger context and objectives to be addressed in the study, and inspired by the results of this initial pilot of the Strong Families programme in Afghanistan.

Although the mental health of children in this pilot study, was assessed by parents only, the inter-rater agreement between parent-, teacher- and self-reports in other studies with similar age groups is a reassurance of the rating of the parents [43, 44].

There was no difference in child behaviour or parenting skills in people recruited via schools or DTC, however through our opportunistic sampling we might have introduced a recruitment bias. It is possible, that only families who regarded themselves in need for support, attended the programme, and hence were more willing to make changes in their behavior. On the other side, people with severe problems might not have the time or understanding of the benefits of such a programme, and hence we might have only recruited families who were eager on showing their good intentions and who might not have had a particular need. However, the parental ratings of their children at baseline indicated a wide range of scores varying between the four bands of the total SDQ score. Nevertheless, the mean score was elevated (high band category of the total SDQ [33]). To overcome this possible bias in either direction in the future, we will follow a strict recruitment protocol, and report on non-responders/non-willing persons thoroughly in our upcoming impact assessment and outcome evaluation measured through well-defined performance indicators and outcome measures.

Family skills programmes in general have been recommended as primary prevention measures as they are more beneficial and hence cost-effective than treatment of possible mental health or substance use disorders [42, 45, 46]. Patel et al. estimated that cost-effective interventions for key mental, neurological, and substance use disorders in low and lower-middle-income countries lies around 3–4 US$per head of population per year, often taking a chronic or disabling course, resulting in additional costs for treatment, with often less than 1% of health expenditure spent on these people.

Family skills interventions have been recommended for multiple primary prevention purposes, such as drug use, interpersonal violence (including against children), by the UNODC WHO International Standards on Drug Use Prevention [42], by the WHO Violence Prevention Alliance for Prevention of Youth Violence [47], by the INSPIRE Interagency Initiative to end violence against children [48] and most recently in a new UNODC Training Manual on Prevention of Child Recruitment and Exploitation by Terrorist and Violent Extremist Groups [49]. Hence, investment in such prevention intervention has shown to be effective and cost-effective [42], particularly in support of several targets of the Sustainable Development Goals.

Recognizing parenting as a key factor for improving outcomes and mitigating children’s exposure to low resource induced risk and harm is also important. Similarly, these results also induces parents living in challenged settings to further engage in their social role with the family and improve care, monitoring, communication, reciprocal support, particularly given the difficult living conditions they are living in.

The results reflected in this pilot study seem to support the aforementioned objectives and as such should encourage adoption of such initiatives by policy makers. In this respect, on a political level, the Strong Families programme also had a significant impact, resulting in the CCPCJ (Commission on Crime Prevention and Criminal Justice “Strengthening the engagement of all members of society in crime prevention”) resolution following the presentation of the data in 2019 [50].

## Conclusions

Based on the findings of our pilot, the implementation of a family skills programme is feasible in a low-resource and challenged setting and family skills can be strengthened. We have shown that light programmes such as Strong Families that can be delivered by lay and trained facilitators. Future research needs to be added to assess the long-term impact of the programme and to compare children with or without the intervention through a thorough outcome and impact evaluation. Our initial results will hopefully motivate policy makers to allow for such an evaluation and integrate such programmes into their countries implementation strategies for reducing negative health and social outcomes. Stronger families can make an impact on youth and supports reaching healthy and safe development of youth particularly those living in such challenged settings.

## Data Availability

The datasets generated and analysed during the current pilot study are available in the Mendeley Data repository, 10.17632/v5dryspfy4.1

## Abbreviations

MOPH: Ministry of Public Health
MCN: Ministry of Counter Narcotics
MOE: Ministry of Education
NGO: Non-governmental organisation
UNODC: United Nations Office on Drugs and Crime
DTC: Drug Treatment Centre
DDR: Drug demand reduction
SDQ: Strengths and Difficulties Questionnaire
PAFAS: Parenting and Family Adjustment Scales
FBQ: Family Background Questionnaire
UK: United Kingdom
US-INL: Bureau of International Narcotics and Law Enforcement Affairs
SD: Standard deviation
PTSD: Post-traumatic stress disorder
SDG: Sustainable Development Goal
WHO: World Health Organization
